# Preliminary results of absorbable magnesium stent for treating eustachian tube dysfunction in a porcine model

**DOI:** 10.1371/journal.pone.0284584

**Published:** 2023-04-25

**Authors:** Jeon Min Kang, Song Hee Kim, Dae Sung Ryu, Yubeen Park, Dong-Sung Won, Ji Won Kim, Chu Hui Zeng, Jung-Hoon Park, Hong Ju Park

**Affiliations:** 1 Biomedical Engineering Research Center, Asan Institute for Life Sciences, Asan Medical Center, Seoul, Republic of Korea; 2 Department of Otorhinolaryngology-Head and Neck Surgery, Asan Medical Center, University of Ulsan College of Medicine, Seoul, Republic of Korea; Shanghai Jiao Tong University Medical School Affiliated Ruijin Hospital, CHINA

## Abstract

Absorbable magnesium (Mg) stents have an attractive biocompatibility and rapid degradation rate, but their degradable behavior and efficacy in the Eustachian tube (ET) have not yet been investigated. In this study, the degradable behavior of the Mg stent in artificial nasal mucus was evaluated. The Mg stents in the porcine ET model were also investigated to evaluate their safety and efficacy. Four Mg stents were placed into the four ETs of two pigs. The mass loss rate of the Mg stents gradually decreased over time. The decrease rates were 30.96% at one week, 49.00% at two weeks, and 71.80% at four weeks. On the basis of histological findings, the thickness of submucosal tissue hyperplasia and the degree of inflammatory cell infiltration significantly decreased at four weeks compared with two weeks. Biodegradation of the Mg stent occurred before tissue proliferative reactions, and the ET patency was successfully maintained without stent-induced tissue hyperplasia at four weeks. The Mg stent that biodegrades rapidly seems to be effective and safe in porcine ET. Further investigation is required to verify the optimal stent shape and indwell period in the ET.

## Introduction

Eustachian tube dysfunction (ETD) is a common disorder in otolaryngology practice that occurs when the mucosal lining of the tube fails to open properly [1]. It interferes with the functions of the ET, such as ventilation and secretion transport into the nasopharynx, and can result in acute or chronic otitis media [2]. Since Ockermann et al. [3] first described balloon Eustachian tuboplasty, an interventional procedure that uses an inflated balloon catheter to restore the ET patency, many studies have reported it as an effective and safe treatment for the ETD over the past decade [4–6]. Nevertheless, given the 36–80% success rate of balloon Eustachian tuboplasty, ET stent placement may be an effective alternative for those who failed to respond to balloon dilatation or if restenosis develops [7–9].

Several preclinical studies have investigated the ET stent placement [10–14]. The technical feasibility and safety of cobalt-chrome alloy stent placement *via* a minimally invasive approach under endoscopic guidance have been reported. However, stent-induced tissue hyperplasia caused by mechanical injuries remains a significant obstacle to a successful stent placement [10, 11]. Drug-eluting stents loaded with antiproliferative agents have been investigated to overcome these limitations. Recently, a sirolimus-eluting stent successfully prevented stent-induced tissue hyperplasia in the porcine ET model [11, 14].

**Fig 1:**
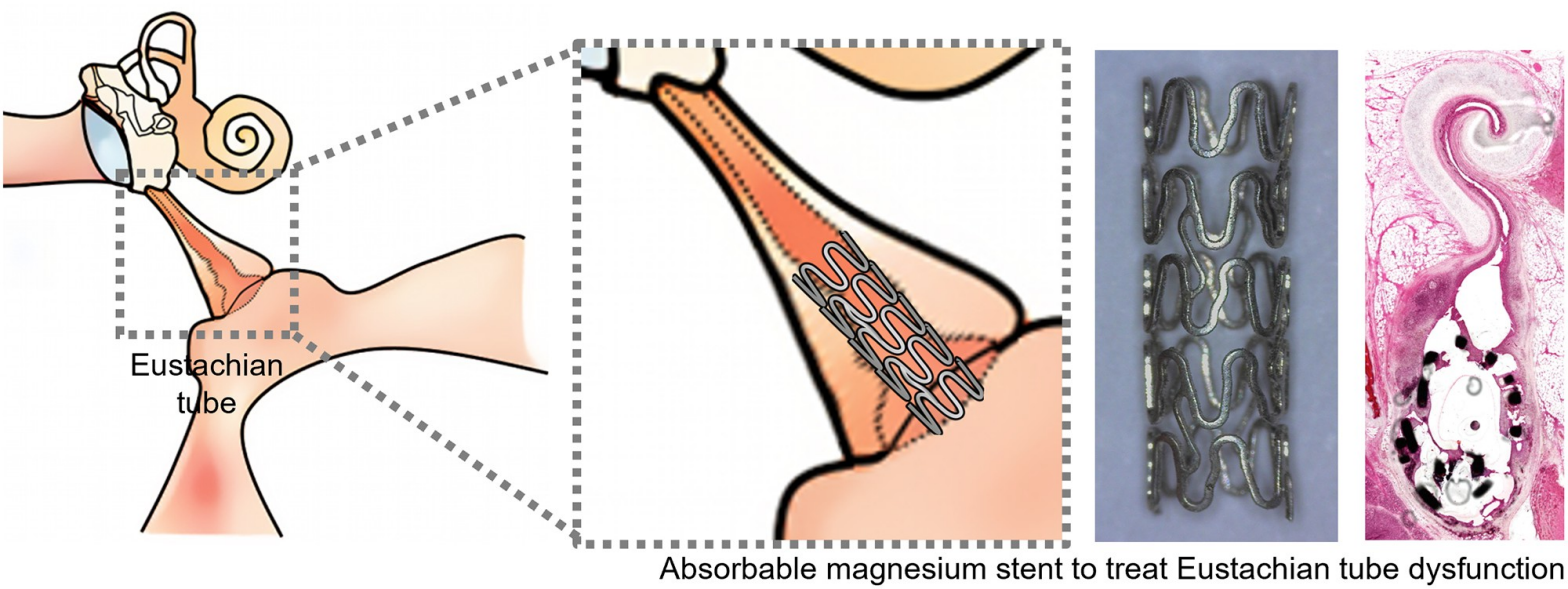
Schematic image of an absorbable magnesium stent to manage ETD showing prevention of stent-induced tissue hyperplasia and maintaining the ET patency. Note. ET, Eustachian tube; ETD, ET dysfunction.

Absorbable magnesium (Mg) stents have attracted more attention to treat vascular and non-vascular obstructions for their fascinating biocompatibility and biodegradability [15, 16]. Regardless of these characteristics, the most significant limitation that prevents the Mg stents from being used clinically is the extremely high rate of degradation within four weeks under physiological conditions [17–19]. However, the faster degradation rate of the Mg stent may be of great utility in the ETD. The Mg stents are thought to be biodegradable in the short term to maintain the ET patency, and they rapidly biodegrade before stent-induced tissue hyperplasia occurs. The purpose of this study was to evaluate the safety and efficacy of Mg stents in the porcine ET by investigating their degradable behavior and luminal patency (Fig 1).

## Materials and methods

### Stents preparation

**Fig 2:**
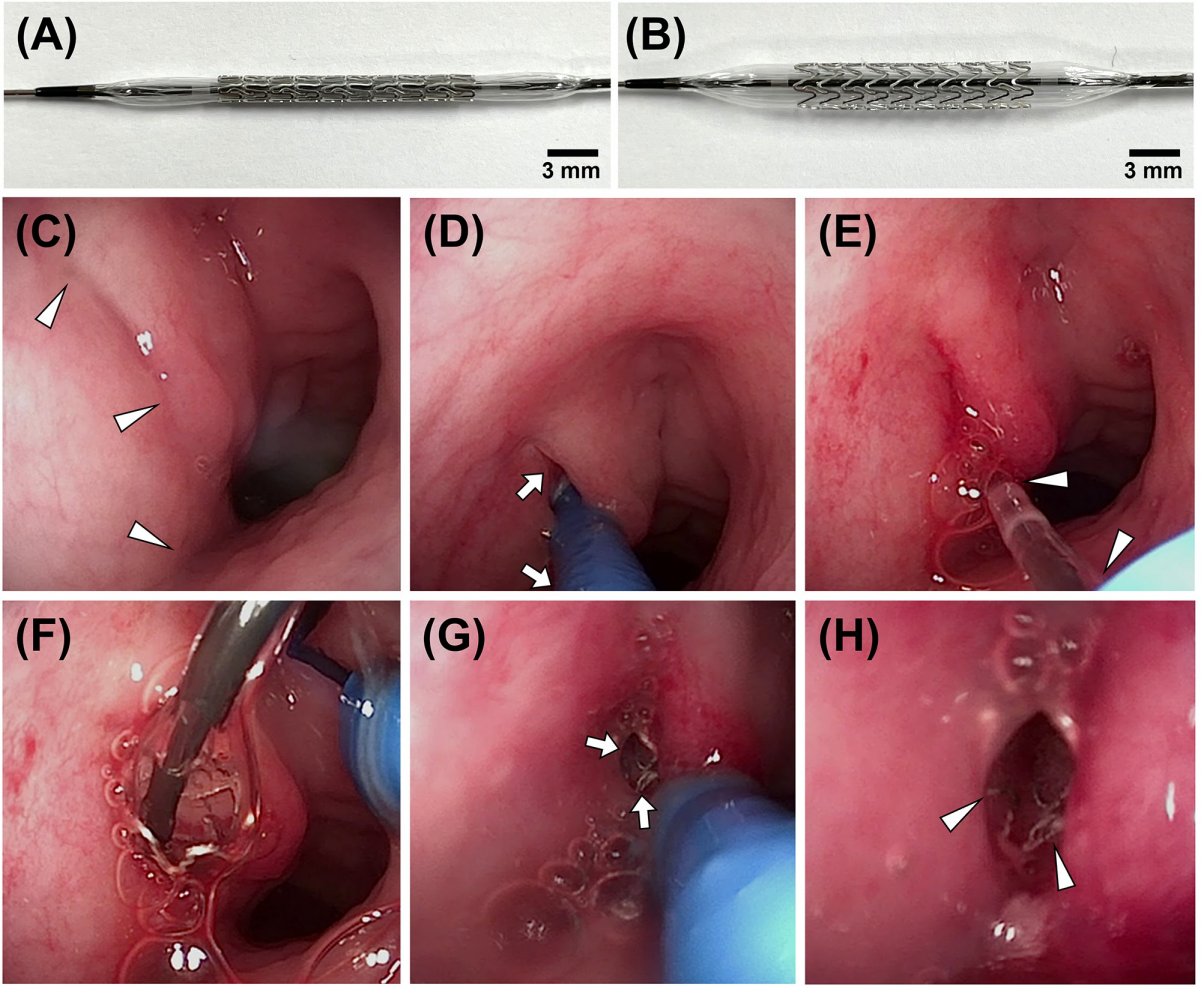
The Mg stent and the technical steps of ET stent placement under endoscopic guidance. Photographs showing (A) the Mg stent loaded onto a balloon catheter and (B) when fully expanded. (C) Endoscopic image showing the nasopharyngeal ostium (*arrowheads*). (D) The steerable guiding sheath (*arrows*) was inserted into the nasopharyngeal ostium. (E) The balloon catheter crimped with the Mg stent (*arrowheads*) is advanced into the ET. (F) The balloon catheter was fully inflated with saline. (G) After verifying the fully expanded stent (*arrows*), the sheath with the balloon catheter was removed. (H) An endoscopic image of the Mg stent (*arrowheads*) in the porcine ET. Note. Mg, magnesium; ET, Eustachian tube.

The absorbable metallic stent made of WE43 Mg alloy (4.1 wt% yttrium, 2.1 wt% neodymium, 0.56 wt% zirconium, 0.028 wt% manganese, and balance Mg, Vascotube, Germany) was designed and manufactured by Genoss Co, Ltd. (Suwon, Korea). The stent had a diameter of 3 mm, length of 16 mm, and strut thickness of 70 μm. The Mg stent was crimped onto a balloon catheter with a diameter of 3 mm and a length of 18 mm (Genoss Co, Ltd) (Fig 2A and 2B). The stents with the same diameter and 8 mm length were used to evaluate degradable behavior.

### Degradable behavior test

A total of 16 Mg stents were placed into a 1.8 ml cryo-tube filled with artificial nasal mucus (BZ253, BioChemazone, Alberta, Canada) at 37°C, respectively. The artificial nasal mucus was tested in a human ET like environment. The surface morphologies of the Mg stents and mass changes were analyzed at one, two, and four weeks, each with four samples. The remaining four Mg stents were used as a control. The Mg stents were removed from the cryo-tube and washed in an ultrasonic bath immersed in isopropyl alcohol. The mass of the washed stents was measured using an electronic balance (Ohaus Corp., Pine Brook, NJ, USA), and the percentage of mass loss was calculated as follows:

$$Mass\;loss\;\left(\%\right)=[\left(M-M_{d}\right)/M\;\times 100]$$

where M and M_d_ were the initial and remaining masses of the stent, respectively [20]. The surface morphologies of the Mg stents were examined using a scanning electron microscope (SEM, Sigma-300, Carl Zeiss, Germany).

### Animal study

The protocol and animals of this study were approved by the Institutional Animal Care and Use Committee of the Asan Institute for Life Sciences (Seoul, Korea) and adhered to the US National Institutes of Health guidelines for humane laboratory animal handling and reported by ARRIVE guidelines (IACUC-2020-12-189). A total of four ETs of two Yorkshire pigs (International Animal Experiment Center, Pocheon, Korea) weighing a mean of 35.3 kg underwent Mg stent placement. One pig at two weeks and one at four weeks after stent placement were sacrificed by intravenously injecting potassium chloride (DAI HAN PHARM CO., Seoul, Korea). All of the pigs were supplied with water and food *ad libitum* and were maintained at a temperature of 24 ± 2°C with a 12-hour day-night cycle.

### Absorbable Mg stent placement under endoscopic guidance

Prior to the ET stent placement, all of the pigs were anesthetized using a mixture of 50 mg/kg zolazepam, 50 mg/kg tiletamine (Zoletil 50; Virbac, Carros, France), and 10 mg/kg xylazine (Rompun; Bayer HealthCare, Leverkusen, Germany). An endotracheal tube was then inserted, and anesthesia was administered by inhalation of 0.5–2% isoflurane (Ifran^®^; Hana Pharm. Co., Seoul, Korea) with 1:1 oxygen (510 mL/kg per minute). An endoscope (CMOS Video-Rhino-Laryngoscope, KARL STORZ, Tuttlingen, Germany) was carefully advanced through the nostril to localize the nasopharyngeal ostium of the ET (Fig 2C). The steerable guiding sheath (OSYMED. Co., Ltd, Yongin, Korea) was placed in front of the nasopharyngeal ostium with the guidance of endoscopy. By pulling the steering controller, the proximal tip of the sheath was bent and then inserted into the ET orifice (Fig 2D). A balloon catheter, which was a crimped Mg stent, was advanced through the sheath into the ET until its tip met the isthmus portion (Fig 2E). The balloon catheter was fully inflated with saline to 9 atm (Fig 2F). The balloon catheter was then deflated, and the sheath with the balloon catheter was carefully removed (Fig 2G). The nasopharyngeal ostium with the placed Mg stent was observed for any procedure-related complications and the location of the proximal end of the Mg stent by post-procedural endoscopic examination (Fig 2H). All pigs underwent an endoscopic examination before and immediately after stent placement and before sacrifice to evaluate the ET patency and the presence of any secretions around the stent [11].

### Computed tomography

The stented ET tissue samples were extracted and then examined using a microcomputer tomography (CT; Skyscan 1173; Bruker-CT, Kontich, Belgium). CT scans were obtained in the axial plane with a slice thickness of 0.5 mm. The CT data was reconstructed using RadiAnt DICOM viewer (version 1.1.20, Medixant Company, Poznan, Poland) to evaluate the residual Mg stent in the ET tissue samples.

### Histological examination

The stent ET samples were fixed in 10% neutral-buffered formalin for three days and then embedded in resin blocks [11]. The resin blocks were transversely sectioned into the proximal and distal portions of the ET sample. The slides were stained with hematoxylin and eosin. The histological evaluations using hematoxylin and eosin staining included the thickness of submucosal tissue hyperplasia and the degree of inflammatory cell infiltration. The degree of inflammatory cell infiltration is determined by the distribution and density of inflammatory cells with the following scores: 1, mild; 2, mild to moderate; 3, moderate; 4, moderate to severe; and 5, severe [21]. The average values for the thickness of submucosal tissue hyperplasia and the degree of inflammatory cell infiltration were obtained from eight points around the circumference. Measurements were obtained using the CaseViewer software (CaseViewer; 3D HISTECH Ltd., Budapest, Hungary). The histological findings were based on the agreement of three observers who were blinded to the study.

### Statistical analysis

The data are expressed as a mean ± standard deviation (SD). Differences between groups were analyzed using the Mann-Whitney U test using SPSS software (version 24.0; SPSS, Inc., Chicago, IL). The *p*-value of < 0.05 was considered statistically significant.

## Results

### Degradable behaviors of the Mg stent

**Fig 3:**
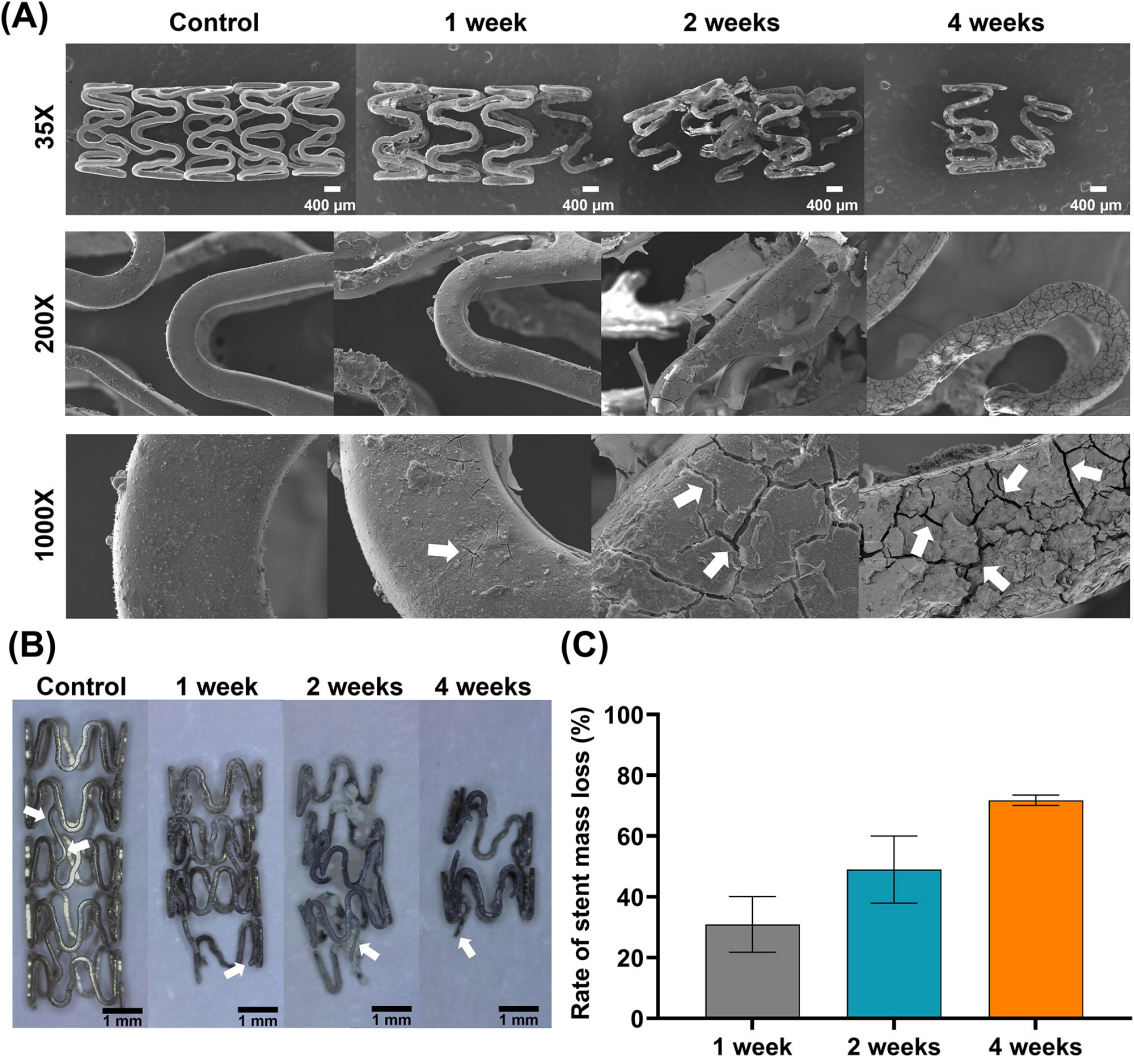
Surface morphologies of the Mg stent and mass loss rates. (A) SEM images showing the size and number of the cracks increasing over time (*arrows*) on the surface of Mg stent samples obtained at one, two, and four weeks, respectively. (B) The bridges (*arrows*) between the struts are disconnected, and the strut was sequentially separated. (C) A graph depicting the mass loss rates of the Mg stent over a four-week period. Note. Mg, magnesium; SEM, scanning electron microscopy.

The percentages of mass loss decreased gradually over time (Fig 3A). The decrease rates were 30.96 ± 5.76% at one week, 49.00 ± 6.93% at two weeks, and 71.80 ± 1.07% at four weeks, respectively. After one week, SEM images revealed that the surface of the Mg stent had microcracks. The size and number of the cracks increased over time. The Mg stent structure was not maintained, the bridges between the struts were disconnected, and the strut was sequentially separated after two weeks (Fig 3B). A mass loss of > 70% of the total stent mass was observed, and severe cracks were also observed at the residual stent struts at four weeks (Fig 3C).

### Procedural outcomes and endoscopic findings

**Fig 4:**
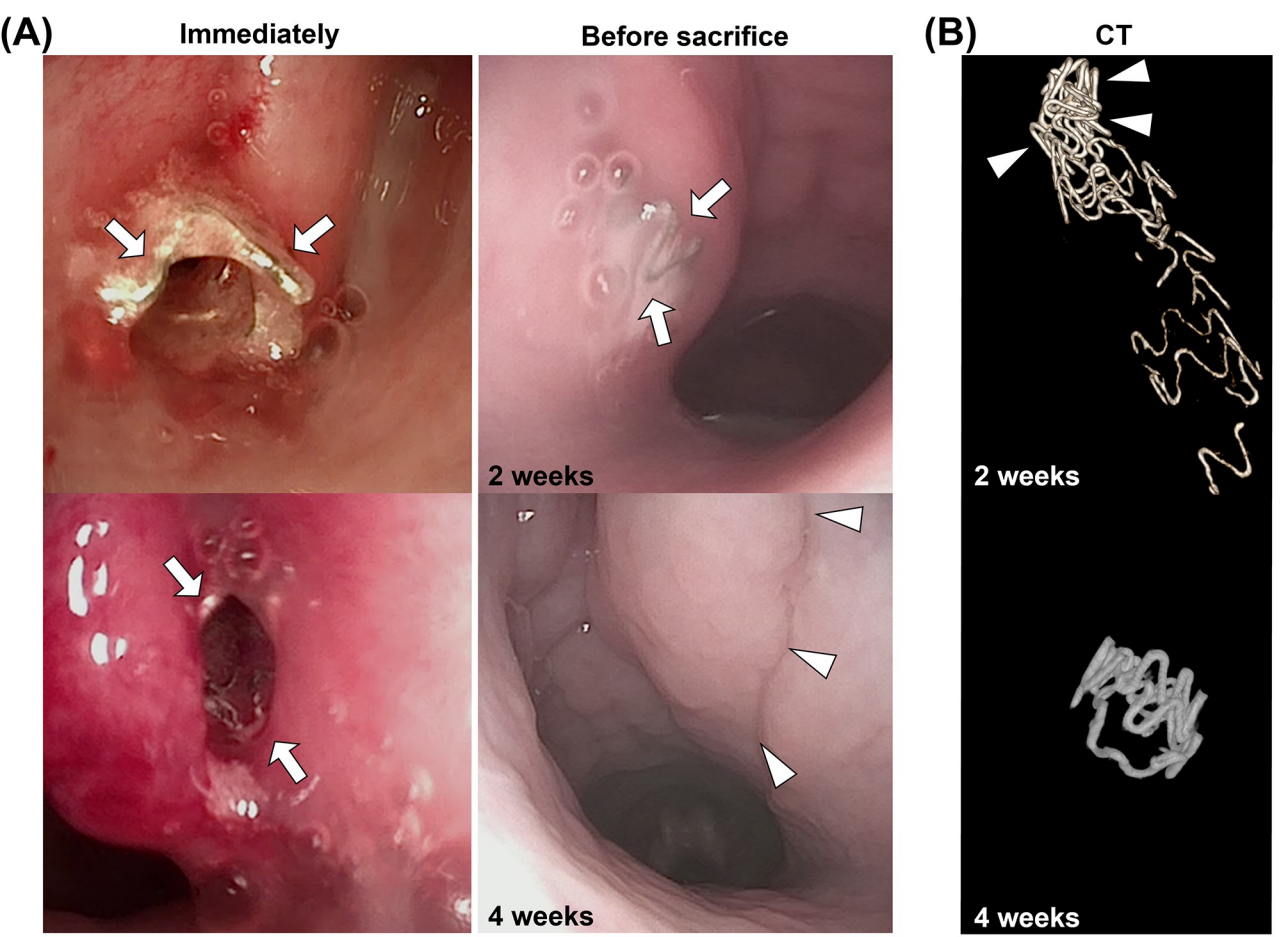
Representative endoscopic and CT images of the stented ET over time. (A) Endoscopic images taken immediately after stent placement, showing the proximal end of the Mg stent (*arrows*) in the porcine ET. The proximal end of the stent with mild secretion (*arrows*) is observed around the Mg stent at two weeks, while the Mg stent is not observed at four weeks (*arrowheads*). (B) 3D-reconstructed CT images of the degraded Mg stent showing a residual Mg stent with a collapsed distal end at two weeks and only a small piece of the Mg stent at four weeks. Note. CT, computed tomography; Mg, magnesium; ET, Eustachian tube.

The Mg stent placement was technically successful in all the porcine ETs without any stent-related complications. The steerable guiding sheath with the Mg stent-crimped balloon catheter was successfully inserted into the nasopharyngeal ostium (Fig 4A). A two-week follow-up endoscopic examination revealed the proximal end of the placed Mg stent. However, the residual Mg stents were not observed at four weeks after stent placement. Mild secretions accumulated around the stents after two weeks, but no secretions were observed after four weeks.

### Computed tomography

Two weeks after stent placement, 3D-reconstructed CT images revealed the residual Mg stent with a collapsed distal end (Fig 4B). Only the residual Mg stent was visible after four weeks. The mean residual volume of the Mg stent was 8.53 ± 1.39 mm^3^ and 4.50 ± 1.15 mm^3^ after two and four weeks, respectively.

### Histological findings

**Fig 5:**
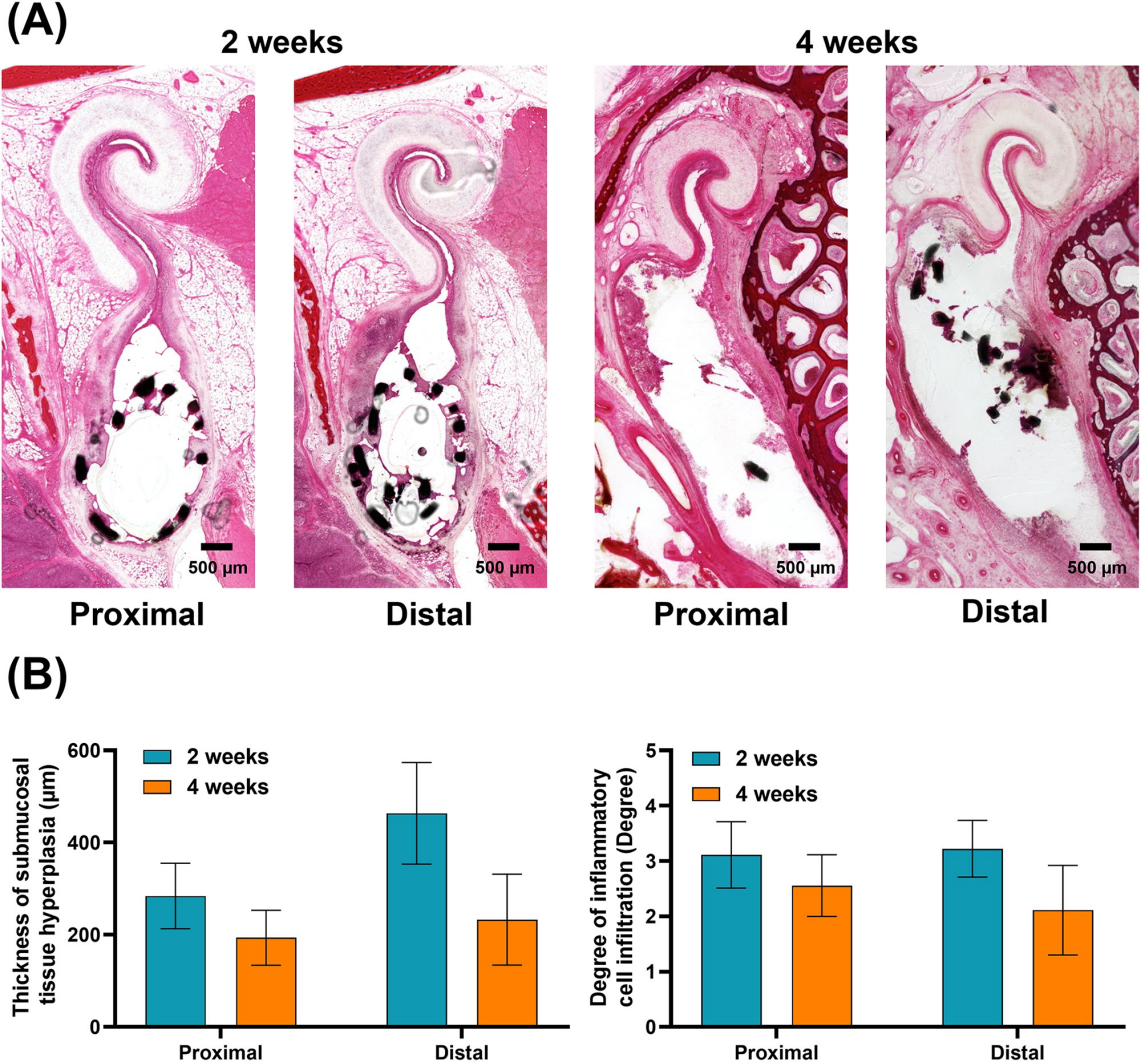
Representative histological images of the porcine ET and histological findings. (A) Histological images showing the submucosal tissue hyperplasia increased at two weeks and then significantly decreased at four weeks. (B) Histological results of the thickness of submucosal tissue hyperplasia and the degree of inflammatory cell infiltration in the proximal and distal portions of the stented ET over time after the Mg stent placement. Note. Mg, magnesium; ET, Eustachian tube.

The histological findings are shown in Fig 5. The mean thickness of submucosal tissue hyperplasia at two weeks was significantly higher than those at four weeks at both the proximal and distal portions of the Mg stent (proximal portion, 284.04 ± 92.25 μm vs. 193.48 ± 77.45 μm, *p* = 0.038, at two and four weeks, respectively; and distal portion, 463.52 ± 143.55 μm vs.232.76 ± 128.02 μm, *p* = 0.002, at two and four weeks, respectively). The distal portion of the Mg stent was significantly higher than that in the proximal portion at two weeks (*p* = 0.006), but there was no significant difference between the portions at four weeks (*p* = 0.442). The degree of inflammatory cell infiltration was not significantly different in proximal portions over time (3.11 ± 0.78 at two weeks vs. 2.56 ± 0.72 at four weeks, *p* = 0.138). However, the distal portion at two weeks (3.22 ± 0.67) was significantly higher than that at four weeks (2.11 ± 1.05; *p* = 0.017).

## Discussion

Absorbable Mg stents are attracting attention for the treatment of vascular and non-vascular stenosis due to their high biocompatibility and non-toxicity [22, 23]. The Mg-based stent was rapidly degraded and lost approximately 97% of its mass within 10 days in vessels [19]. In the current study, the degradable behavior of the Mg stent in an environment (artificial nasal mucus) similar to the ET was investigated. Micro-cracks were observed on the surface of the Mg stent after one week. The size and number of the cracks significantly increased over time, and 70% of the mass of the stent was lost at four weeks. When compared to the vessels, the Mg stents degraded more slowly in the ET. The bridge connecting the struts was degraded preferentially, and the struts were sequentially lost while maintaining the original shape of the stent even if the cracks increased on the stent surface. Although it has a rapid degradation period compared to other biodegradable stents, the application of the Mg stents to the ET stent placement seems to be useful as a temporary solution for the ETD.

Stent-induced tissue hyperplasia was significantly evident two weeks after stent placement in the porcine ET model due to the mechanical pressure of the stents [10, 11]. The ET continuously functions with opening and closing movements; thus, long-term stent placement would be limited. In our study, although the optimal indwell period of stent placement in the ET has not been sufficiently investigated, stent-induced tissue hyperplasia was significantly reduced compared with previous studies using non-degradable stents, including cobalt-chrome alloy and self-expandable nitinol stents [10, 11, 24]. Tissue hyperplasia proliferates through the wound healing cascade caused by stent-mediated mechanical injuries [25, 26]. At two weeks, stent-induced tissue hyperplasia was observed in the ET but it was confirmed without tissue remodeling at four weeks. In addition, the residual Mg stents remaining in the ET lumen and residual struts being absorbed from the submucosal layer were observed. The residual Mg stent could not be confirmed using endoscopic findings, but we assumed that the residual Mg stents could drain into the nasopharynx with nasal mucus. The Mg stent placed in the porcine ET was rapidly decomposed within two weeks, and the mechanical force of the Mg stent was also gradually decreased.

The ET consists of two portions and is tapered towards the middle ear. The first portion is a relatively wide tube surrounded by cartilage, while the second is a narrow isthmus portion surrounded by bone [27]. This cartilaginous portion is used for stent placement in the ET [28]. The absorbable Mg stent is a balloon expandable stent that has no self-expanding ability, and it expands based on the shape of the inflated balloon. The distal portion of the Mg stent in the ET could not maintain its round shape. Previous studies reported that the cobalt-chrome alloy stent for the porcine ET also failed to keep a round shape [10, 11]. The thickness of submucosal tissue hyperplasia and the degree of inflammatory infiltration at two weeks were significantly higher in the distal portion than in the proximal portion of the stent. The ET has a morphological structure that gradually narrows from the nasopharynx to the middle ear. Our histological findings demonstrated that relatively small diameter of the distal portion of the ET has severe submucosal tissue hyperplasia and inflammation after stent placement compared with the proximal portion of the ET. Mild mucus accumulation was observed in the endoscopic findings at two weeks, and no mucus was observed around the ET at four weeks. The used Mg stent induced tissue hyperplasia and inflammation reactions in the porcine ET, but the Mg stent was degraded over time and these tissue reactions gradually decreased. The potential efficacy of the Mg stent has been verified but the development of an optimized shape for the ET is still required.

This study has some limitations. First, the Mg stent was placed into the normal porcine ET. Additional studies should be conducted using an ETD animal model. Second, we did not evaluate changes of radial force of the Mg stent because the stent samples were easily broken from the first week. Third, we also did not investigate the long-term follow-up analysis after the complete decomposition of the Mg stent. Fourth, we did not compare the Mg stent to the nonbiodegradable stent in terms of tissue reactions following stent placement. Fifth, the number of animals was too small. Further study with a large number of animals is required to verify our findings. Finally, the optimal period of stent placement in the ET has not been verified. Even though the Mg stent was degraded within four weeks and tissue hyperplasia was reduced, the optimal period of placement requires investigation.

## Conclusion

Biodegradation of the Mg stent occurred within two weeks before an actively proliferative tissue reaction while ET patency was successfully maintained without stent-induced tissue hyperplasia at four weeks. The Mg stent that biodegrades rapidly seems to be effective and safe in porcine ET. Placement of the Mg stent could be an alternative therapeutic option for refractory ETD after balloon Eustachian tuboplasty. The temporary Mg stent placement can eliminate the chance of stent-induced tissue hyperplasia formation and stent removal, while maintaining the ET patency within two weeks. Even though further preclinical studies are required to verify the optimal stent shape and indwell period in the ET, the application of the Mg stent in the ET has the therapeutic potential for ETD.
